# Pharmacogenetics of asparaginase in acute lymphoblastic leukemia

**DOI:** 10.20517/cdr.2018.24

**Published:** 2019-06-19

**Authors:** Rachid Abaji, Maja Krajinovic

**Affiliations:** ^1^Charles Bruneau Cancer Centre, Research Center, CHU Sainte-Justine, University of Montreal, Montreal, QC H3T 1C5, Canada.; ^2^Department of Pharmacology & Physiology, University of Montreal, Montreal, QC H3C 3J7, Canada.; ^3^Department of Pediatrics, University of Montreal, Montreal, QC H3T 1C5, Canada.

**Keywords:** Asparaginase, pharmacogenomics, hypersensitivity reactions, pancreatitis, relapse, acute lymphoblastic leukemia, adverse drug reactions

## Abstract

Asparaginase is a key component in leukemias and lymphomas treatment protocols and is suggested as a treatment for other malignancies in which an amino acid depletion strategy is indicated. Asparaginase intolerance is subject to inter-individual variability and can manifest as hypersensitivity reactions, pancreatitis, thrombosis, as well as metabolic abnormalities, and may affect treatment outcome. Pharmacogenetics aims at enhancing treatment efficacy and safety by better understanding the genetic basis of variability and its effect on the pharmacological responses. Many groups tried to tackle the pharmacogenetics of asparaginase but the potential implementation of such findings remains debatable. In this review, we highlight the most important findings reported in studies of the pharmacogenetics of asparaginase related complications and treatment outcome in acute lymphoblastic leukemia.

## Asparaginase and acute lymphoblastic leukemia

*L*-asparaginase (ASNase) is a key component in leukemias and lymphomas treatment strategies and is universally incorporated into major childhood acute lymphoblastic leukemia (ALL) treatment protocols^[1-4]^. ALL in adults has a much lower incidence than in children, and poor survival rates in this population pose a significant challenge^[5]^. The incorporation of ASNase into adults and young adult protocols is still limited due to its toxicity profile in this population^[6]^. On the other hand, the introduction of ASNase into pediatric regimens for ALL treatment and the intensification of its use, along with dexamethasone and vincristine (VCR), is to be credited for most of the improvement in ALL treatment outcome^[1]^. A typical ALL treatment protocol consists of phases that focus on remission-induction, consolidation and maintenance. ASNase is usually administered during the induction phase as well as throughout the consolidation therapy where it is administered for 20-30 weeks together with glucocorticoids and vincristine^[5,7]^.

ALL accounts for 30% of pediatric cancers and is the most common childhood malignancy in developed and underdeveloped countries^[1,8,9]^. The past few decades have witnessed a revolution in the treatment of ALL as survival rates increased considerably from less than 40% in the mid-sixties to currently exceed 90% for most international protocols^[1,2,10-12]^. This result was achieved by the creation and continuous optimization of multi-agent protocols through evidence based medicine, refined stratification of patients into risk groups, personalized chemotherapy that exploit the differences in the characteristics between host and leukemia cells, and improvement in supportive care^[5,12-14]^. While these figures seem quite encouraging, there is a large margin for improvement as treatment failure, cancer relapse, and treatment-related toxicities continue to jeopardize the lives of a significant percentage of children with ALL^[8]^. It is estimated that almost 50% of patients will experience at least one acute severe toxicity, and that a considerable percentage of mortality among leukemia patients is attributable to adverse-events of the treatment rather than the actual sickness^[2,12,15]^. In fact, these toxicities can often be life-threatening and are the primary cause of interruption or discontinuation of chemotherapy^[10]^ and are a frequent cause of sequelae on the long-term^[2]^. Indeed, the recent improvement in survival rate has resulted in a gradual shift towards putting more focus on reducing the toxicity burden of chemotherapy^[2,15]^.

Consequently, several research groups are investigating biomarkers that can predict the risk of treatment resistance or treatment-related adverse effects even before starting the therapy in the hope of being able to modify the treatment in a patient-tailored manner that would increase the probability of response and reduce the risk of side-effects. This is the core goal of pharmacogenetics (PGx) which aims at enhancing treatment efficacy and safety by providing a better understanding of the genetic basis of variability and its effect on the pharmacological responses^[14,16]^. Indeed, there are several success stories in which PGx discoveries have restructured the medical practice and the classical example is the genotyping of *TPMT* gene to guide the dosing of mercaptopurine which is considered mandatory in almost all recent practice guideline^[17]^. Accordingly, many groups studied the pharmacogenetics of ASNase aiming to uncover the genes mediating ASNase antileukemia effect and the genetic basis of interpatient variability in response. However, the implementation of such findings in ALL management remains debatable. In this review, we highlight the most important findings reported up-to-date which tackled the PGx of ASNase-related complications and treatment outcome. We used different search-engine tools - but mainly the ones embedded in the NCBI platform- to identify eligible scientific papers that included the word asparaginase along with either the term pharmacogenomics or pharmacogenetics. Upon evaluating the content of these papers, a filtering step was applied in order to retain only the articles that specifically addressed the PGx of ASNase, which are summarized in Table 1.

**Table 1:** This table summarizes the prominent studies in the literature which investigated the pharmacogenetics of asparaginase and highlights the most important finding

Study	Method	Gene	Polymorphism	Toxicity	Discovery cohort (*n*)	Internal validation cohort (*n*)	Validated	Notes and conclusions
Chen *et al*.^[25]^ 2010	GWAS	*GRIA1*	rs4958351	HSRs	322	163	Yes	rs4958351 had the strongest association in the original study (Chen *et al*.^[25]^ 2010). Carriers of the minor allele were at increased risk of developing HSRs to ASNase. The associations of the variants with increased risk of HSRs found in the original study were successfully replicated by (Rajic *et al*.^[31]^ 2015, *n* = 146) but not by (Kutszegi *et al*.^[9]^ 2015, *n* = 505)
Rajic *et al*.^[31]^ 2015	Gene-Candidate		rs10070447					
			rs6890057					
Kutszegi *et al*.^[9]^ 2015	Gene-Candidate		rs4958676 *rs6889909*					
Fernandez *et al*.^[4]^ 2014	Gene-Candidate	*HLA-DRB1*	*HLA-DRB1* *07:01	HSRs	541	1329	Yes	The variant allele was associated with an increased risk of ASNase HSR
								Alleles that confer high-affinity binding to ASNase epitopes contribute to the observed higher frequency of HSRs
Fernandez *et al*.^[30]^ 2015	GWAS	*HLA-DRB1*	rs17885382	HSRs	3308	No	N/A	The variant allele was associated with an increased risk of ASNase induced HSR and is in almost perfect linkage disequilibrium with HLA-DRB1*07:01 found in a previous study
		*NFATC2*	rs6021191					The variant is associated with an increased risk of ASNase HSR
Ben Tanfous *et al*.^[10]^ 2015	Gene-Candidate	*ASNS*	rs3832526	Allergy	285	248	Yes	Patients homozygous for the triple repeat allele (*3R*) had the complications more frequently than other genotype groups
				Pancreatitis			No	ASNS haplotype *1 harbouring double repeat (*2R*) allele conferred a protective effect from these toxicities
Abaji *et al*.^[41]^ 2017	EWAS	*MYBBP1A*	rs3809849	Pancreatitis	302	282	Yes	This variant was also associated with allergy, thrombosis, event-free survival and overall survival
		*IL16*	rs11556218				Yes	This variant was also associated with thrombosis
		*SPEF2*	rs34708521				Yes	This variant was also associated with thrombosis
Wolthers *et al*.^[15]^ 2017	GWAS	*ULK2*	rs281366	Pancreatitis	700	No	N/A	14 of the 27 associations found in the study were of polymorphisms in *ULK2* gene
		*RGS6*	rs17179470					The variant in *RGS6* gene was associated with pancreatitis in patients less than 10 years old The risk of pancreatitis associated with carrying the risk alleles of rs281366 and rs17179470 was additive in patients less than 10 years old
Liu *et al*.^[35]^ 2016	GWAS	*CPA2*	Gene-Level	Pancreatitis	5,185	213	Yes	16 SNPs in this gene were significantly associated with pancreatitis in a gene-level analysis and around 54% of carriers of at least one of these polymorphisms developed the toxicity. rs199695765 showed the strongest association
Alachkar *et al*.^[6]^ 2017	Gene-Candidate	*SOD2*	rs4880	Hepato-toxicity	190	No	N/A	Increased risk of hepatotoxicity following ASNase-based treatment for carriers of the minor allele
Rousseau *et al*.^[18]^ 2011	Gene-Candidate	*ATF5*	rs11554772	EFS	318	267	Yes	Carriers of the minor allele who received *E. coli* ASNase were at higher risk of ALL relapse and the result was corroborated through replication study and higher promoter activity
		*ASNS*	*rs3832526*					Homozygous carriers of the double repeat (2R) had significantly lower EFS
Pastorczak *et al*.^[54]^ 2014	Gene-Candidate	*ASNS*	*rs3832526*	EFS/Response	264	No	N/A	Carriers of the (3R) allele with a poor response at day 15 had an increased risk of events

ALL: acute lymphoblastic leukemia; ASNase: asparaginase; ASNS: asparagine synthetase; EFS: event-free survival; EWAS: exome-wide association study; GWAS: genome-wide association study; HSRs: hypersensitivity reactions; N/A: not applicable; SNPs: single-nucleotide polymorphisms; *E. coli: Escherichia coli*

## Mechanism of action, resistance and formulations

**Figure 1:**
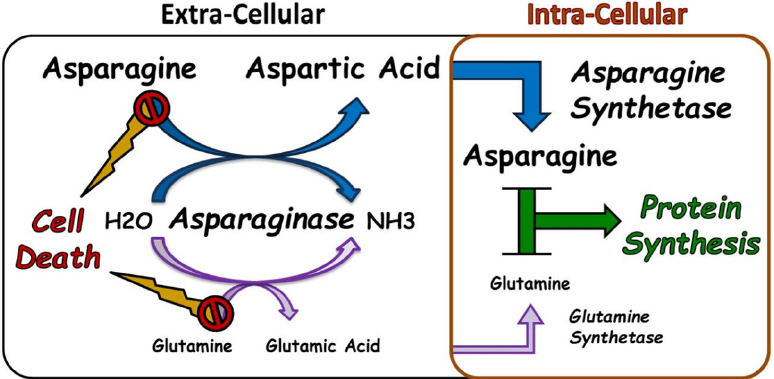
Mechanism of action of Asparginase. Illustration of the mechanism of action of asparaginase as an anti-leukemic agent. The activity of asparaginase leading to the depletion of extra-cellular asparagine and/or glutamine and eventual cell death is counteracted by the intra-cellular production of these amino acids through asparagine synthetase and glutamine synthetase, respectively. To be noted: the relative difference in size between the Asparagine and Glutamine Pathways is meant to reflect the magnitude of the effect of Asparaginase on amino acide depletion which is more prominenet in the context of the asparagine compared to glutamine

The exact mechanism of the anti-leukemic effect of ASNase is still not fully understood. However, it is generally accepted that this enzyme works by hydrolysing asparagine - and glutamine - in the serum, thus depleting the extracellular compartment from these amino acids essential for survival of all cells [Figure 1]^[7,10,14,18,19]^. Asparagine is produced by the enzyme asparagine synthetase, encoded by the *ASNS* gene, which catalyzes the transfer of an amino group to aspartic acid to form asparagine, and may thus counteract the effect of asparaginase and produce resistance as suggested by *in vitro* experiments conducted in leukemia cell lines and patient lymphoblasts^[5,10,18]^.

It has been hypothesized that malignant lymphoblasts have low expression of the *ASNS* gene, or alternatively, are incapable of upregulating the expression of *ASNS* when exposed to ASNase or nutritive stress; subsequently making them unable to produce enough asparagine or glutamine to meet the high demand required for their rapid growth. This renders the leukemic cells more dependent on extracellular sources of asparagine and thus more sensitive to the effect of ASNase which hence selectively kills them by depleting the media of asparagine, leading to amino acid starvation and disrupting the biosynthesis of proteins and eventually cellular apoptosis and death^[2,7,10,14,18,19]^.

As for glutamine, ASNase-resistant lymphoma cells were demonstrated to have a substantial increase in glutamine synthetase activity compared to ASNase-sensitive cells - consequently increasing their production of glutamine; and thus, their proliferation capacity was less affected by low levels of extracellular glutamine^[20]^. Moreover, it was also shown that the transport of glutamine into the ASNase-resistant cells was significantly elevated due to an adaptive-regulation response^[20]^. Furthermore, in a study that evaluated the effect of ASNase on glutamine-dependant lymphoid cell lines, the authors reported a relationship between cells’ sensitivity and the expression pattern of molecules involved in glutamine and asparagine metabolism^[21]^.

The *in vitro* and *in vivo* sensitivity to ASNase have been associated with childhood ALL prognosis^[14,19]^. Inter-individual differences in *ASNS* expression levels and ALL sensitivity to ASNase were noted, which might be explained by a change in expression of *ASNS* gene itself, or genes coding for the regulators of its expression [e.g., The basic region leucine zipper activating transcription factor 5 (*ATF5*); and arginosuccinate synthase 1 (*ASS1*)]. Nonetheless, the body of evidence reporting on the associations between *ASNS* activity and ASNase resistance is conflicting^[5,14,18,19]^. Other causes of resistance include the formation of ASNase inactivating antibodies, the secretion of asparagine from mesenchymal cells in the bone marrow, or altered expression in genes involved in apoptosis^[14,19]^. A study that tested almost 2.4 million SNPs in a genome-wide association study (GWAS) approach using the HapMap lymphoblastoid cell-line, identified aspartate metabolic pathway as a contributor to ASNase sensitivity with primary involvement of *ADSL* and *DARS* genes. The authors were also able to reproduce significant associations in primary ALL leukemic blasts^[19]^.

Historically, three asparaginase preparations were commercially available and each of them has different pharmacokinetic properties. The original preparation was derived from *Escherichia coli* (and is referred to as *E. coli* asparaginase), but it has been abandoned by most developed countries due to its toxicity profile (particularly allergic reactions), and the adoption of its less immunogenic pegylated form (PEG-asparaginase). While PEG-asparaginase is relatively more expensive than its parent-compound, it is considered to be a safer and more effective treatment with a prolonged duration of activity. The third product is a formulation derived from *Erwinia chrysanthemi* (*Erwinia* asparaginase) and is generally associated with lower immunogenic properties and less toxicity. However, its pharmacokinetic profile was reported to be associated with poorer treatment outcome when compared to other formulations at a similar posology (mainly attributed to its shorter duration of activity), suggesting the need for higher doses and increased frequency of administration in order to achieve optimal asparagine depletion. Thus, its use is usually restricted to patients who develop allergic reactions “or silent inactivation” to the *E. coli*/PEG-asparaginase owing to the lack of cross-reactivity^[1,5,7,8,10,22]^; although it is important to mention that controversies on ASNase antibodies formation and its activity have been reported^[23]^. Several clinical trials have reported associations between success of ALL treatment and ASNase dose intensity or formulation^[10,22]^. Of note, enzyme variants with reduced *L*-glutaminase coactivity are being tested for their clinical utility as antileukemic agents with potentially lower side effects (since several studies suggested that the depletion of *L*-glutamine may correlate with many of the side effects of the enzyme). For example, a recent study demonstrated that novel low *L*-glutaminase variants derived from modifications to Erwinia asparaginase can offer high efficacy against both T-Cell and B-Cell ALL while provoking less toxicities^[24]^.

## Pharmacogenetics of asparaginase

### Hypersensitivity reactions, pancreatitis and thrombosis

Since ASNase is a foreign protein produced in bacteria, it is not surprising that all formulations of ASNase, to varying extents, have the immunogenic potential to provoke the formation of antibodies which can either be associated with clinical symptoms manifested in ASNase allergy and hypersensitivity reactions (HSRs), or can be asymptomatic but still capable of neutralizing the activity of ASNase leading to suboptimal response and thus referred to as “silent hypersensitivity” or “silent inactivation” which occurs in up to 30% of patients^[1,8,9,12,25]^. While the allergic symptoms can be mitigated through premedication with anti-histamines and corticosteroids, this still does not prevent ASNase inactivation^[12]^. It is important to mention that higher systemic exposure to ASNase was associated with a lower clearance of dexamethasone, and thus a higher systemic exposure and an increased risk of osteonecrosis. Nonetheless, studies also found that the formation of ASNase antibodies can increase the systemic clearance of dexamethasone, consequently reducing its serum levels and increasing the risk of Central Nervous System relapse^[1,26-28]^.

HSRs are the most common side-effect and can manifest as pain around the injection site, urticaria, flushing, fever, chills, dyspnea, bronchospasm edema/angioedema, and hypotension. They could arguably occur in as much as 75% of patients and could manifest as life-threatening anaphylactic reactions in 10% of them and usually require changing the drug formulation^[2,5,7-10,12,18,25,29]^. The incidence is dependent on different factors which include the number of doses received, route of administration, type of formulation used, re-challenging after a period of interruption, and the administration of concomitant medications during the course of treatment^[1,8,9,12,25,30]^.

One of the pioneer studies in the context of PGx of HSRs was a GWAS which aimed at identifying germline genetic polymorphisms that could contribute to the risk of allergy in an ethnically diverse population of 485 ALL children treated with ASNase on St. Jude Children’s Research Hospital treatment protocol Total Therapy XV. They interrogated over 500,000 single nucleotide polymorphisms (SNPs) and had many significant hits. Essentially, the results demonstrated an overrepresentation of SNPs in genes located on chromosome 5q33 in general (which is already known to be associated with several inflammatory or autoimmune diseases), and in the *GALNT10* and *GRIA1* genes in particular. Indeed, the associations of five of the polymorphisms (i.e., rs4958381, rs10070447, rs6890057, rs4958676, and rs6889909) in *GRIA1* gene with HSRs were successfully validated within the same study in an independent replication cohort^[25]^ and were later replicated in an independent Slovenian population of 146 pediatric ALL patients mainly treated according to one of Berlin-Frankfurt-Münster (BFM) treatment protocols^[31]^. Moreover, the authors reported an association between the frequency of ASNase allergy and racial ancestry; with patients of Caucasian origins developing allergic reactions at a higher frequency than those of Black or Hispanic ones^[25]^. Another group tried to replicate the results by targeting 20 SNPs in *GRIA1* and *GALNT10* genes in a candidate-gene fashion in a group of Hungarian ALL children treated as part of the BFM Study Group. Briefly, they were unable to replicate any of the results in the total cohort. However, interestingly, they found an opposite association between rs4958381 in *GRIA1* and reduced risk of HSRs in the T-cell ALL subgroup but not in the pre-B-cell ALL patients. Moreover, they reported significant associations of two SNPs in *GRIA1* not identified in the original work (but only in the medium risk group), which can still serve as a further evidence of the implication of the *GRIA1* gene in the modulation of the risk of ASNase induced HSRs and might suggest that the influence can vary depending on subgroups^[9]^.

In another study that involved a total of 1870 patients of European ancestry, the authors imputed human leukocyte antigen (HLA) alleles and searched for significant associations with ASNase hypersensitivity in childhood ALL patients from Jude Children’s Research Hospital and the Children’s Oncology Group. They reported a strong association of HLA-DRB1*07:01 allele in both groups and demonstrated that HLA-DRB1 alleles that confer high-affinity binding to ASNase epitopes contribute to the observed higher frequency of HSRs^[4]^.

Another GWAS was performed on a cohort of 3,308 pediatric ALL patients treated according to St. Jude Children’s Research Hospital protocols or Children’s Oncology Group protocols and demonstrated that variants within genes regulating the immune response, particularly genes involved in T-cell function, strongly influenced the risk of ASNase hypersensitivity. The authors found a strong association between a polymorphism in the nuclear factor of activated T cells 2 (NFATC2), rs6021191, and hypersensitivity to ASNase. They also reported that the association was strongest among patients receiving native *E. coli* ASNase as compared to PEG-ASNase and that carrier-status of this intronic variant was associated with a higher expression of the gene’s messenger RNA compared to noncarriers (both in ALL leukemic blast samples and lymphoblastoid cell-lines). Moreover, looking at the association of nonsynonymous coding variants with HSRs, they found that the most significant association was that of rs17885382 in *HLA-DRB1* which is in almost perfect linkage disequilibrium with HLA-DRB1*07:01 mentioned earlier and can be used as a confirmation of the importance of the latter in influencing the risk of ASNase hypersensitivity. Importantly, this finding extends the role of the polymorphism to non-European patients; since the new cohort was ethnically diversified as opposed to the previous one which only involved patients with European ancestry. Furthermore, the authors also demonstrated that the risk of HSRs associated with carrying the risk alleles of rs6021191 in *NFATC2* and rs17885382 in *HLA-DRB1* was additive^[30]^.

In a study performed on samples from 359 Hungarian childhood ALL patients treated with one of the BFM protocols and aimed at using next-generation sequencing to identify associations between ASNase hypersensitivity and polymorphisms of the Human Leukocyte Antigen (HLA) Class II region alleles, the authors further confirmed that variations in HLA-D region can influence the development of ASNase HSRs. For example, patients with HLA-DRB1*07:01 allele or HLA-DQB1*02:02 allele had a significantly higher risk of developing this toxicity compared to non-carriers. Moreover, a significant association with the haplotype HLA-DRB1*07:01-HLA-DQB1*02:02 was observed as carriers of this haplotype were at higher risk than carriers of only one of the risk alleles. Furthermore, carrying the HLA-DRB1*07:01-HLA-DQA1*02:01-HLA-DQB1*02:02 haplotype was associated with the highest risk of ASNase hypersensitivity. Of note, this study also reported that HLA-DQB1*02:02 allele was significantly less frequent in the proportion of patients with T-cell ALL than in pre-B-cell ALL patients^[32]^.

Since patients with PEG-asparaginase HSRs were demonstrated to have no ASNase enzymatic activity, a more recent study investigated genetic predisposition to PEG-asparaginase hypersensitivity in a GWAS analysis by defining the hypersensitivity phenotype as both having clinical hypersensitivity and no enzymatic activity. The genetic analysis was performed on fifty-nine cases and 772 control pediatric patients treated on the Nordic Society of Paediatric Haematology and Oncology (NOPHO) ALL2008 protocol. The study found rs73062673 polymorphism of the *CNOT3* gene to be associated with PEG-asparaginase allergy. Of note, this gene was previously shown to regulate the transcription of HLA and to act as a tumour suppressor which is frequently mutated in T-cell ALL. The study also reported the detection of two other associations involving rs9272131 and rs115360810 variants in the HLA-DQA1 and TAP2 genes, respectively. While these associations were not significant on a genome-wide level, they remain of a particular interest since the variants are located in a region known to be highly involved in allergic responses. These results further suggest the implication of genetic variations in the HLA region, as well as regulators of these genes, in the mechanisms leading to asparaginase hypersensitivity^[33]^.

Other common adverse-events to ASNase are acute pancreatitis and cerebrovascular accidents, such as thrombosis, which can occur in 18% and 5% of ALL patients, respectively; and are usually dose limiting^[2,10,12,15,18]^. Pancreatitis symptoms can range from being mild and self-resolving, to a more severe systemic inflammatory response syndrome and failure of pancreatic function^[15]^. While the risk of mortality due to ASNase induced acute pancreatitis is relatively low, the risk of recurrence upon re-challenge is almost 50% and patients affected by it have a higher risk of developing chronic or relapsing pancreatitis as well as acute or persistent diabetes mellitus^[12,15,34]^. Clinical factors of ASNase associated pancreatitis include Native American ancestry, older age, and higher cumulative ASNase exposure^[35]^. While the role of genetics in predisposition to acute recurrent and/or chronic pancreatitis of different etiologies has been the focus of many studies (PRSS1, PRSS2, SPINK1, CFTR, CLDN2, CAP1)^[34,36-39]^, ASNase-related acute pancreatitis have only started emerging recently.

In a work that tackled the PGx of ASNase through candidate-gene approach, by the association between SNPs in *ASNS*, *ATF5* and *ASS1* genes and ASNase induced allergy and pancreatitis was investigated in a discovery cohort of 285 ALL patients and a replication cohort of 248 patients who were treated according to Dana-Farber Cancer Institute ALL Consortium protocols. The authors reported a significant association between a 14-bp tandem-repeat polymorphism *rs3832526* in *ASNS* gene and both of these toxicities as patients homozygous for the triple repeat allele (*3R*) had the complications more frequently than other genotype groups. Moreover, when analysing the effect of possible haplotypes, they found that the *ASNS* haplotype *1 harbouring double repeat (*2R*) allele conferred a protective effect from these toxicities and its association with allergy was further validated in an independent replication cohort. Furthermore, they showed that one of the subtypes of this haplotype was associated with reduced *in vitro* sensitivity to ASNase in lymphoblastoid cell lines^[10]^.

It is worth mentioning that in a study including 472 Japanese children with ALL who were treated on a protocol that included *E. coli* derived asparaginase, the authors followed a candidate-gene approach aimed at replicating the associations found with *GRIA1* rs4958351, *NFATC2* rs6021191, and *ANSN* rs3832526. The authors reported no significant association between any of the variants and HSRs which suggests that the role of these variants might be influenced by ethnic specific differences in genetic structure surrounding them^[40]^.

Another work followed an exome-wide association study approach which was performed on 302 children with ALL treated according to DFCI protocols and the results were validated in an independent group of 282 patients following protocols of the same institution. The authors interrogated around 4.5 thousand SNPS distributed across 3,802 genes and reported 12 associations with ASNase complications in the discovery cohort including 3 with allergy, 3 with pancreatitis and 6 with thrombosis along, with a strong additive effect of combining more than one polymorphism. Interestingly, rs3809849 in the *MYBBP1A* gene was associated with allergy, pancreatitis, thrombosis, event-free survival (EFS) and overall survival, while rs11556218 in *IL16* gene and rs34708521 in *SPEF2* gene were both associated with thrombosis and pancreatitis. Importantly, the association of each of these three polymorphisms with pancreatitis was replicated in the validation cohort^[41]^. Of note, our search results could not identify other original research work that investigated the PGx of ASNase-induced thrombosis, which could be an interesting field for future studies.

In a GWAS study of ASNase-induced pancreatitis that involved ALL patients treated following St Jude Children’s Research Hospital and the Children’s Oncology Group protocols, the discovery group was composed of 5,185 children and young adults with ALL and was replicated in an independent case-control group of 213 patients. While the authors reported no significant association of common variants at the GWAS level, they detected a significant association for a rare nonsense variant rs199695765 in *CPA2* gene. Interestingly, in a subsequent gene-level investigation, 16 SNPs in this gene were significantly associated with pancreatitis with around 54% of carriers of at least one of these polymorphisms ended up developing it^[35]^.

In another GWAS study of 700 children who were treated following the NOPHO ALL2008 protocol, the authors interrogated around 1.5 million SNPs and found 27 significant associations with ASNase related pancreatitis. rs281366 variant in *ULK2* gene showed the strongest association with pancreatitis, and interestingly, 14 of the 27 associations were of polymorphisms in this same gene. In a sub-analysis focusing on patients who were less than 10 years old, rs17179470 in *RGS6* was strongly associated with pancreatitis. Moreover, in this particular subgroup, more than half of the cases carried one of these two risk alleles and the risk of pancreatitis associated with carrying both alleles was additive. Of noteworthy, *ULK2* gene is involved in autophagy, and *RGS6* regulates G-protein signaling regulating cell dynamics^[15]^.

In a larger and more recent multi-centric study lead by researchers from the same group, the authors investigated the risk of ASNase-associated pancreatitis in a discovery cohort of 244 cases and 1320 controls through GWAS analysis^[15]^. rs62228256, a variant located in a noncoding region of the genome upstream from the *NFATC2* gene, and for which it acts as an expression quantitative trait loci (eQTL) in pancreatic tissue, had the strongest association signal with an increased risk of pancreatitis for carriers of the minor allele. However, the validation analysis in a cohort of 33 cases and 285 controls who followed one of the DFCI treatment protocols did not replicate this association. An association with pancreatitis was also detected for minor alleles of rs13228878 and rs10273639 which reside on the same haplotype and are in high linkage disequilibrium within the *PRSS1-PRSS2* locus encoding for cationic and anionic trypsinogen, respectively. The association was further confirmed in a replication analysis performed on samples from patients of the Children’s Oncology Group (76 cases and 2,653 controls). Of note, these variants were associated with an increase in the expression of *PRSS1* gene and they have been previously linked to alcohol-associated and sporadic pancreatitis in adults. Another interesting outcome of this study is the further validation of the association between pancreatitis risk and SNPs within genes known to regulate trypsin activation. Accordingly, minor alleles of rs17107315 in pancreatic secretory trypsin inhibitor (*SPINK1*), rs10436957 in chymotrypsin C (*CTRC*), and rs4409525 in Claudin-2 (*CLDN2*) all had significant associations with modulating the risk of ASNase-induced acute pancreatitis with directions and effects similar to the previously reported findings. The authors also applied a targeted genotyping approach to test the reproducibility of the association of the *ULK2* variant rs281366 and *RGS6* variant rs17179470 with the risk of pancreatitis previously reported by the same group but the results were not significant^[15]^.

### Other less common toxicities

ASNase intolerance can also result in hepatotoxicity, abnormalities of hemostasis, hyperglycemia, hyperlipidemia, and may also affect treatment outcome since it was shown that patients who experienced a dose-limiting ASNase toxicity had a significantly worsened disease-free survival^[2,8,10,12,18]^. However, due to the rarity of these toxicities, they were less frequently investigated. ASNase-induced hepatotoxicity is one of the most common ASNase complications in adults treated for ALL but is rarely investigated in genetic studies since most of such studies focus on the use of ASNase in pediatric patients. Given its mechanism of action, ASNase induces amino acid stress response by depleting asparagine and glutamine. This results in an excessive production of reactive oxygen species (ROS) and a subsequent increase in mitochondrial permeabilization and eventual cell apoptosis; a process that has been linked to ASNase-induced hepatotoxicity^[6]^. In a candidate-gene analysis that involved 190 adult ALL patients enrolled on CALGB-10102, the authors reported a significant association between homozygous carriers of the minor allele of rs4880 in *SOD2* gene, a mitochondrial enzyme that protects cells against ROS, and an increased risk of hepatotoxicity following ASNase-based treatment^[6]^.

### Relapse

Relapse is a major cause of treatment failure in pediatric ALL as it was reported to arise in 11%-36% of patients with high-risk B-precursor ALL^[42-49]^. The risk of relapse and treatment toxicity can be modulated by multiple factors, and differences in genetic composition among patients have recently driven considerable attention^[5,16]^. Several PGx studies reported that genomic variation was associated with higher risk of relapse in ALL patients^[42,50,51]^. For example, in a GWAS that involved 2,535 children with newly diagnosed ALL that aimed at targeting germline polymorphisms associated with relapse, the authors identified 5 SNPs linked to higher levels of ASNase antibodies and 2 of those were associated with a higher relapse rate^[51]^. In a more recent study that investigated the contribution of germline genetic factors to relapse in 2,225 children treated according to the Children’s Oncology Group trial AALL0232 protocol, the author reported that the group of relapse SNPs in the more ASNase intensive treatment arm was overrepresented with SNPs linked to ASNase resistance or allergy^[42]^.

Early reports have indicated that lower exposure to ASNase during ALL treatment can result in an increased risk of relapse^[52,53]^, which lead a research team to hypothesising that genetic polymorphisms of genes in asparagine pathway (i.e., *ASNS*, *ATF5*, and *ASS1*) can be associated with risk of event-free survival and relapse leading to a study that involved 318 Caucasian children with ALL and an independent replication cohort of 267 patients^[18]^. Indeed, the authors identified a variant in the promotor of *ATF5* gene, rs11554772, and a higher risk of ALL relapse in patients who received *E. coli* ASNase. This gene codes for a transcriptional factor involved in *ASNS* gene regulation. Importantly, the result was validated in the replication group and was corroborated with data on the association of the same polymorphism with higher promoter activity. Another finding was the association of a 14-bp tandem-repeat polymorphism, *rs3832526*, located in the first intron of *ASNS* gene and EFS which showed that homozygous carriers of the double repeat (2R) had a significantly lower EFS, but the association lacked significance in the validation cohort^[18]^. They also reported the association of polymorphisms in the *ASS1* gene and EFS, albeit these associations did not sustain correction for multiple testing and thus were not further investigated in the replication cohort^[18]^. Interestingly, the repeat polymorphism in *ASNS* gene was later linked to early response to ALL treatment following the administration of a single ASNase dose in a study of 264 Polish children with ALL. However, the association was in the opposite direction as carriers of the (3R) allele with a poor response at day 15 had an increased risk of events, hence the data suggest an interaction between this polymorphism and early response to treatment that could result in variability of EFS rates^[54]^.

### MicroRNA

One area that is currently under-investigated in relation to the effect of ASNase is that of microRNAs (miRNA), with only few studies reporting associations between differences in miRNAs expression levels and response in childhood ALL^[55-57]^. While reports suggest that the expression of over 60% of protein coding genes is subject to regulation via miRNAs^[58]^. Of note, many groups linked the expression levels of specific miRNAs to clinical outcome of ALL patients. In fact, studies suggest that miRNA expression profiles can differ significantly between ALL genetic-subtypes and that drug-resistant cases are associated with unique miRNA signature. For example, one study showed that miR-454 was expressed at nearly two-fold lower levels in ASNase-resistant pediatric ALL patients when compared to ASNase-sensitive ones^[55]^. Another study linked miR-210 to ASNase-sensitivity as demonstrated by the expression levels dependent change in the minimum inhibitory concentration (IC_50_), the concentration needed to block the proliferation of half of the initial cell population^[56]^.

## Conclusion

While it is becoming increasingly recognized that both tumor and germline genomics can influence response to treatment, the latter is less commonly used to guide treatment in oncology settings. It should be emphasized that the possibility of detecting a random signal in association studies is relatively high, which could explain the conflicting data and inconclusive results among studies that targeted the same genetic-phenotypic associations. Moreover, differences in trial settings, treatment protocols, nature of supportive care, the degree of scrutiny with which an outcomes is measured and variations in disease characteristics, among others, can influence the role of the variant in question^[10,12]^. One example is the leukemic cells that carry the subtype of ALL featuring a TEL/AML1 fusion gene which were demonstrated to be more sensitive to the effect of ASNase compared to other subtypes^[59]^. Thus, the implication of a gene or its polymorphisms in the outcome should only be taken into consideration for clinical implementation if the association was confirmed by independent studies and further supported by functional analysis.

The translatability of pharmacogenetics findings into the clinical realm of personalized medicine remains a challenge given the complex interplay between the host and malignancy genomes. One example is the CoALL 06-97 study which incorporated a combined drug resistance profile into their risk group stratification process of 224 patients. While this profile, which was based on *in vitro* cellular resistance to prednisolone, VCR and ASNase, was previously shown to be linked to treatment response and was confirmed in several studies, the authors reported no significant difference between results of that study and those of historical control group stratified according to conventional risk factors^[60]^. A lot of work needs to be done in the context of implementation of pharmacogenetics. In a study that analyzed pharmacogenomics literature of 125 drugs used in oncology, more than half of the drugs (55%) did not have pharmacogenomics data while only 12 of those which did, had actionable associations^[61]^. Understanding the pharmacogenetics of ASNase can help refining treatment strategies for other cancers in which asparagine and/or glutamine depletion can be indicated, such as in subtypes of acute myeloid leukemia, sarcomas, pancreatic and ovarian malignancies^[62-65]^.

Given the recent breakthroughs in biotechnology allowing for increasingly shorter rendering time and lower costs of genotyping and sequencing services, pharmacogenetics will continue to flourish as more complex analyses will be feasible. This will enrich the pool of validated genetic markers that can predict the risk and outcome of a particular treatment and will make it possible to move away from the less-than-optimal trial-and-error approach to dosing, towards the implementation of PGx to guide a treatment that is tailored to the genetics of each individual.

## Declarations

### Copyright

© The Author(s) 2019.
